# Knowledge of Preconception Healthcare and Associated Factors: A Study among Mothers in Jinka Town, Southern Region, Ethiopia

**DOI:** 10.1155/2021/7529805

**Published:** 2021-02-28

**Authors:** Kassahun Fikadu Tesema, Tamirat Cheneka, Alemayehu Alemu, Mekonen Feyissa, Birhanu Birkaye, Hafiza Mohammed, Eleni Kidu, Getahun Wegaso, Biresaw Wasihun

**Affiliations:** ^1^Department of Midwifery, College of Medicine and Health Sciences, Arbaminch University, Arba Minch, Ethiopia; ^2^Maternal and Reproductive Health at Arba Minch University, Midwifery Department, Arba Minch, Ethiopia

## Abstract

**Background:**

Preconception healthcare is promising to improve the reproductive health status of women and couples if they receive care three months to two years before conception. In the current context of Ethiopia, however, preconception healthcare is overlooked in the continuum of care. Therefore, this study aimed to assess the knowledge of preconception healthcare and associated factors: a study among mothers in Jinka town, southern region, Ethiopia.

**Methods:**

A community-based cross-sectional study was employed among 522 randomly selected women of childbearing age who are living in Jinka town from March to April 2018. The study considers all the kebeles in the town. Study subjects were determined using proportionate-to-population size allocation. Then, a systematic random sampling technique was applied. Data were collected using a semistructured and pretested questionnaire. Descriptive summary data and binary logistic regression analysis were carried out to identify factors with the 95% confidence level and a *p* value of less than 0.05.

**Results:**

A total of 513 study subjects participated in this study. The overall preconception healthcare knowledge score of women in Jinka town was 51.1%. In the multivariable analysis, housewives (AOR = 2.93; 95% CI: 1.38–6.19), an education level of at least college (AOR = 3.79; 95% CI: 1.75–8.23), no history of neonatal death (AOR = 4.13; 95% CI = 1.39–12.25), and the use of family planning methods (AOR = 2.38; 95% CI: 1.49–3.79) increased the probability of preconception healthcare knowledge compared to the counterparts.

**Conclusion:**

In this study, women's knowledge of preconception healthcare was found borderline. The identified factors were housewife, education level of at least college, no history of neonatal death, and using family planning methods. Therefore, emphasizing these factors for the enhancement of women's knowledge of preconception healthcare is a necessary step.

## 1. Introduction

Preconception care aims to enhance health before conception by facilitating risk screening, health promotion, and effective interventions as a part of routine healthcare [1]. Besides, it has a high impact if all reproductive age couples become involved, whether or not they are contemplating pregnancy [2]. Most girls become pregnant before they know that they are pregnant [3]. In most low-income settings, however, maternal healthcare does not start until pregnancy is well established, not until quite half of the pregnancy has passed [4]. Others enter into the pregnant state without having optimal health status while practising risky health behaviours; all imply that there is a gap in the continuum of care given to childbearing women [1, 5].

There is growing evidence that shows the implementation of preconception care initiatives in low- and middle-income countries (LMIC) [5–8]. Due to the implementation of preconception healthcare initiatives, women and her fetus have a healthy gestation and beyond. Nevertheless, additional evidence is required to see the effectiveness of interventions where the worth of primary service integration is promoted. It is also an economical and socially acceptable way of providing primary preventive look after for all couples of childbearing age who are getting to have a healthy pregnancy outcome [1].

Receiving inadequate or no preconception care increases the magnitude of unplanned pregnancies, during which rates of maternal and infant death rate are linked to a scarcity of access to preconception care [7, 9]. Research has speculated that about 35% of pregnancies with untreated gonococcal infections ended in low birth weight infants or premature deliveries, and up to 10% of this leads to perinatal death [5]. In this regard, the danger of lower birth weight and preterm delivery often increases in subsequent pregnancies [10, 11].

Moreover, women who are not planning a pregnancy, quitting cigarettes smoking, and have a habit of substance use before conception have an increased risk of adverse maternal and child health outcomes [7, 12, 13]. Poor maternal outcomes such as chronic hypertension, preeclampsia, and placental abruption [14, 15] possess an increased risk of adverse birth outcomes [16]. Women's awareness of preconception healthcare, which emphasizes on the preexisting condition of women [17], can reduce the danger of adverse pregnancy outcomes at large [18, 19].

For example, with the implementation of a comparatively simple intervention such as folic acid supplementation, immunization, and lifestyle modifications before pregnancy can reduce adverse pregnancy outcomes [20–22]. During this, cessation of tobacco exposure or elimination of cigarette smoking alone can reduce the typical of 6.5% and 23.5% of preterm delivery and sudden infant death rate, respectively [5].

Studies in low-income countries including Africa revealed low preconception healthcare knowledge that targeted the reproductive age group women. As an example, a study conducted in Malaysia reported that almost 52% of girls who had visited maternity clinics had satisfactory knowledge of preconception healthcare [23]. Another study in Egypt speculated that about 39% of girls attending antenatal care knew the role of folic acid supplementation within the prevention of fetal malformations [24]. Research findings in Nigeria and Sudan also showed that women's knowledge of seeking preconception health interventions was reported very low [25, 26]. Research findings in Ethiopia indicated that mothers who had good knowledge regarding preconception healthcare are low. For instance, a community-based study in Wolayita [27] and Gojjam [28], Ethiopia, showed that 53% and almost 28% of mothers knew preconception healthcare, respectively. Even though it is essential, preconception health is yet not integrated into the present healthcare system in Ethiopia [29, 30]. Hence, preconception healthcare may be a missed opportunity that the maternal healthcare system of Ethiopia should account for [13]. During a remote health setting where the notice of preconception health is low, the promotion of preconception health is, therefore, necessary either to spice up the continuum of care and reduce adverse pregnancy outcomes. Besides, evidence of women's knowledge of preconception health and associated factors in the study area are scarce. This study aimed to assess knowledge of preconception healthcare and associated factors: a study among mothers in Jinka town, southern region, Ethiopia.

## 2. Methods and Materials

### 2.1. Study Setting, Design, and Period

Jinka is an administrative town located 560 km far away from Hawassa and 750 km from Addis Ababa, at latitude and longitude of 5°47′N 36°34′E/5.783°N 36.567°E. The town is an administrative town of South Omo Zone within the Southern Nations, Nationalities and Peoples' Region. Within the year 2018, the entire population estimated was 30493, including 15217 (49.9%) males and 15276 (50.1%) females. Of the entire females, 7103 (23.3%) were in their reproductive age group [31]. Jinka town has one general hospital, two health centres, and 6 health posts. Community-based cross-sectional study was conducted among a randomly selected 522 women from March to April 2018 in Jinka town administration.

### 2.2. Population

All of the mothers within the reproductive age group who live in Jinka town were the target group. Women of the reproductive age group who live in the selected kebeles of Jinka during the study period were the study population. Women of the reproductive age group who live in Jinka town for 6 months were included. During this study, women with hearing problems and important illness were not eligible.

### 2.3. Sample Size Determination and Sampling

The sample size was determined using a single population proportion formula (*n* = *Z*^2^*p* (p−1)/*d*^2^) considering the following assumptions: 27.5% of the proportion of women's knowledge of the previous study in Northern Ethiopia [28], a confidence interval (CI) of 95%, a marginal error (*d* = 4%), and 10% nonresponse rate. Based on this, the final estimated sample size was 527. A simple random sampling technique was applied to select households from six kebeles of Jinka town. The household number was determined using an updated list of the kebeles administrative office. Then, a computer-generated simple random sampling technique was employed. The number of households eligible to be interviewed was estimated using proportional to size allocation. Using proportional to size allocation, the probability of women being selected is proportional to the size of the overall women of the reproductive age group in the selected kebele, giving the kebeles with larger proportion had a greater probability of selection while the smaller one had lower probability. A lottery method was applied when more than one candidate was found per household. Revisiting helps to reduce the number of nonresponses when the interviewee is not present during the data collection day.

## 3. Operational Definition

### 3.1. Knowledge

Women's knowledge of preconception healthcare was measured using thirteen items of preconception healthcare questions.

#### 3.1.1. Good Knowledge

The knowledge index was built from answers to 20 questions on how diabetes mellitus should be treated and controlled before conception [2], epilepsy should be treated before conception [3], uncontrolled obesity affects fetal health [4], screening for STI and HIV/AIDS improves fetal life [5], heart disease should be treated before conception [6], stress before conception affects fetal life [7], screening for a genetic problem before conception lowers adverse pregnancy outcome [8], cigarette smoking should be avoided before conception [9], alcohol consumption before conception results in poor pregnancy outcome [10], exposure to environmental hazards before conception leads to adverse perianal outcome [11], habit of illegal drug intake before conception affects fetal wellbeing [12], routine female genital mutilation complicates childbirth [13], maintaining prepregnancy weight prevents adverse pregnancy outcome [14], avoiding cigarette smoking before conception can be a must [15], avoid alcohol consumption before conception is mandatory [16], tetanus vaccination prevents neonatal infection [17], having regular medical check-up helps to realize health pregnancy outcome [18], getting folic acid and vitamin supplements before conception lowers probability of fetal malformation [19], creating a healthy environment before conception benefits pregnancy [20], and planning pregnancy helps to realize healthy pregnancy outcome. Where a participant answered “yes” or “no” to all or any questions. Based on the answers to those questions, the index knowledge was categorized as having good knowledge (score 10–20).

#### 3.1.2. Poor Knowledge

The knowledge index was built from answers to the 20 questions, and based on the answer of those questions, the knowledge index was categorized as having poor knowledge (score less than 10). A pretested, interviewer-administered, structured data collection instrument was developed adopting from literature based on the study objectives [26, 28, 32, 33]. The questionnaire was divided into data that were collected divided into sociodemographic characteristics, birth outcome events, chronic illness profiles, general awareness of preconception healthcare, and knowledge of preconception healthcare.

The reliability coefficient was computed using SPSS version 26 window-compatible software. The overall across-item reliability coefficient of the instrument was 0.849. On the contrary, content validity was assessed by three independent maternal and child health experts at Arba Minch University, and therefore, the average content validity index score of 86% was noted. Each question had one correct response where those that score above the mean of knowledge measuring questions are labelled as women having good knowledge.

### 3.2. Data Collection and Management

Primarily, the questionnaire was prepared in English and translated into the Amharic version using language experts. Face-to-face interview was conducted using the Amharic version. For data analysis, the Amharic version was reverted to the English version to keep the data consistent and clear. Data collection was conducted using five diploma midwives. The involved data collectors and supervisors were fluent in speaking the local language and had similar community-based experiences. Two BSc midwives who had previous experience were hired for supervision. Completeness and consistency of the questionnaires were checked before, during, and after the data collection period. The principal investigator and the supervisors checked the data for missing values and completeness; corrective measures were taken accordingly. Data collectors and supervisors were trained for 2 days on the questionnaire, data collection procedure, confidentiality, and consent.

### 3.3. Data Analysis and Interpretation

Data were checked manually for consistency and completeness at the site of data collection. Then, the cleaned data were entered into EPI info version 7.2 and exported to a Statistical Package for Social Science (SPSS) version 26 windows compatible software for analysis. Descriptive summary statistics, such as percentage and frequency, were computed and presented in tables and graphs. To identify associated factors with women's knowledge of preconception healthcare, bivariate logistic regression was performed for each independent variable and dependent variable. Independent variables with a *p* value of less than 0.2 in the bivariate analysis were included in the multivariable analysis. A *p* value of <0.05 or 95% confidence interval excluding 1 in the multivariable analysis was considered statistically significant.

## 4. Results

### 4.1. Sociodemographic Characteristics

**Figure 1:**
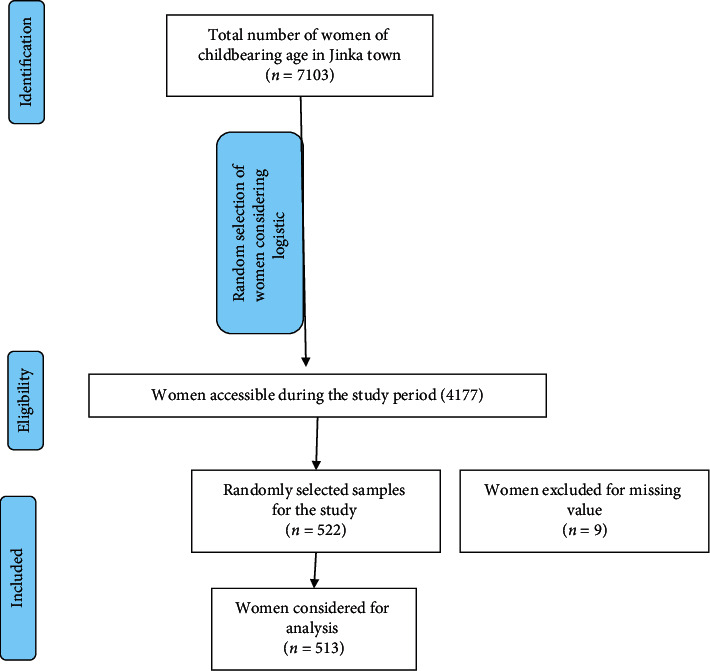
Flow chart for included and excluded study participants in a preconception knowledge assessment study conducted among women of reproductive age living in Jinka town, Southern Ethiopia.

From a total of 522 study subjects, 513 participated in this study, which gives a response rate of 98.7% (Figure 1).

The median age of the respondents was 24 years. Three hundred and thirty-seven (65.7%) of the study participants were married. The majority, 257 (50.1%) of study subjects were Orthodox Tewahido religion followers, while the rest 188 (36.6%) and 62 (12.1%) were protestant and Muslim religion followers, respectively. Nearly sixty percent of the study subjects had Mali ethnic background followed by about 28% of Amhara ethic dwellers. In this study, the percentage of women who have no formal education was 13.3%. The majority of the 208 (40.5%) of them were housewives. Study participants had a proximate record in terms of educational status; 157 (30.6%) of them had achieved primary education level while secondary education level was followed by 155 (30.2%). The majority, 377 (73.5%) of study subjects have used their mobile phones to access health related information, while the rest 241 (46.9%) and 97 (18.9%) were using radio and television. In this study, nearly 8% had no regular access to any of the abovementioned means of communication (Table 1).

### 4.2. Adverse Birth Outcome Events of Reproductive Age Women

Among the 513 respondents, 333 (64.9%) had reported that they were pregnant before. Of the total participants, 34 (10.2%) had an unintended pregnancy history. Of the 71 (21.3%) study participants who had an episode of abortion, 53 (10.3%) of them had completed spontaneously. Regarding the history of poor obstetric events, women who had a stillbirth, neonatal death, preterm birth, and congenital anomaly were 5.4%, 7.5%, and both preterm birth and congenital anomaly had shared 2.10%, respectively.

### 4.3. Chronic Health Illness Profile and Social Behaviour

**Figure 2:**
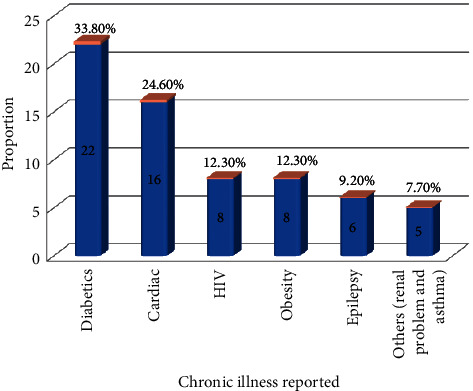
Types of chronic health problems childbearing age women have in Jinka town, Southern Ethiopia, 2018 (*n* = 65).

Of the 513 women, 484 (94.3%) of them drank no alcohol, while 496 (96.7%) of them responded that they were not smoking. Of the total participants, 304 (59.3%) had used at least one family planning method. Regarding the history of existing health conditions, sixty-five (12.7%) women had at least one confirmed chronic illness. In this study, women with diabetics mellitus were 22 (33.8%) (Figure 2).

### 4.4. Women's Awareness of the General Concept of Preconception Care

The overall preconception healthcare knowledge score of women in Jinka town was 51.1%. Among the 513 participants, 312 (60.8%) of them have heard about preconception care before. The most frequently mentioned source of information about preconception healthcare was health institutions, where it accounted for 247 (48.1%). The remaining included mass media (19.55%), family/relatives (15.1%), school, and friends (10.89%) (Table 2).

### 4.5. Women's Knowledge of Preconception Health and Behavioural Risk Factors on the Fetus

Results of women's knowledge of risk factors towards pregnancy outcome: STIs including HIV/AIDS (80.3%), untreated obesity (49.7%), cigarette smoking (86.7%), alcohol consumption (83.0%), female genital mutilation (74.5%), and genetic problems (47.4%) were sexual and behavioural conditions of preconception healthcare (Table 3).

### 4.6. Women's Knowledge of Changes Should Be Made before Pregnancy

**Figure 3:**
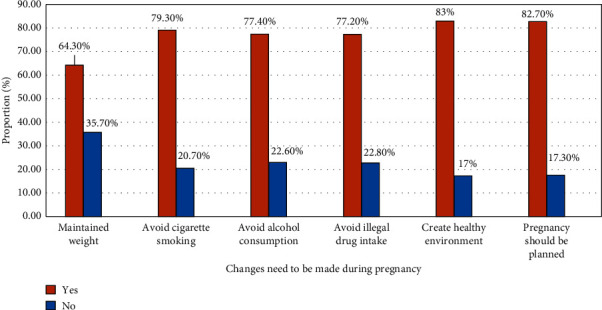
Women's knowledge of preconception care component in Jinka town, Southern Ethiopia, 2018.

Creating a healthy living environment (83%), having a planned pregnancy (82.7%) and medical screening (51.5%), avoiding cigarette smoking (79.3%), and getting a vaccination (66.6%) and folic acid supplementation (30.6%) were conditions where women should be aware before conception (Figure 3).

### 4.7. Factors of Knowledge of Preconception Care

Results from the binary logistic regression models for the association between independent variables and women's preconception healthcare knowledge while controlling covariates are presented in Table 4. The likelihood of having good preconception health knowledge was higher in housewives (AOR = 2.93; 95% CI = 1.38–6.19). Women who had attended primary education (AOR = 2.06; 95% CI = 1.06–3.97), secondary education (AOR = 2.77; 95%CI = 1.38–5.57), and tertiary education (AOR = 3.79; 95%CI = 1.75–8.23) were more likely to have good preconception healthcare knowledge.

Last, the odds of having good preconception healthcare knowledge was higher in women who had no history of neonatal death (AOR = 4.13; 95% CI = 1.39–12.25) and used family planning methods (AOR = 2.38; 95% CI = 1.49–3.79).

## 5. Discussion

In this study, the overall study participants' knowledge of preconception healthcare was found to be 51.1%. The findings in this study are higher than studies conducted in West Gojjam (27.5%) [28], Saud Arabia (37.9%) [33, 34], Nigeria 20.6% [35], India (6%) [36], Egypt (22%) [37], Southeastern Nigeria (43.1%) [38], and Sudan (11.1%) [25]. This may be due to time variation; as time goes, women's understanding becomes better. Furthermore, in the case of the Sudanese and Saudi Arabian studies, a single component of preconception healthcare was addressed. In Gojjam, more than 34% of the study participants had an awareness of preconception healthcare than the 60% counterparts in the current study. In this study, more women (87%) had attended at least primary education than women in Gojjam's does. The participation of rural women may also be a source of heterogeneity, and this study focuses on women living in urban areas.

A significant variation has been shown between this study and studies conducted in Jordan (85%) [39], Iran (68.8%) [40], and the USA (76%) [41]. Women who live in the middle- to high-income countries have better information and Internet access and better media coverage. On the other hand, a gap in creating awareness using public media may exist in the current study.

Indeed, preconception healthcare is not a part of the functioning healthcare system of Ethiopia. In the developed region, the presence of speciality clinics designed to give maternal care given by the accoucheur might enhance the women's interest in preconception healthcare. Indeed, most of the women presented in those clinics might have higher levels of education status.

However, the results reported in this study are relatively similar to the findings in Nepal (49%) and Wolayita (53%), Ethiopia, [27].

### 5.1. Factors of Women's Knowledge of Preconception Healthcare

The result of this study revealed that housewives were more likely to have preconception health knowledge. The current study finding is in agreement with a study conducted in China [42], where women's occupation determines their knowledge level. Housewives may have access to health information from health facilities because they usually bring their children to follow up [43]. Also, these women tend to have time to contact health extensions, friends, and healthcare providers, and Internet use.

This research finding speculated that women's level of education increases their knowledge of preconception healthcare. This is consistent with different studies conducted in Gojjam [28], China [42], Nigeria [35], Sudan [25], and Iran [40]. An educated woman has increased access to preconception care-related information more likely. Education can provide better information access and enhanced critical thinking in which women have access to preconception healthcare. The higher the women's education level, the better she understands preconception healthcare services and has a favourable attitude on similar issues [44]. Scientifically speaking, highly educated people have greater intentions to prepare for their pregnancy [45].

This study showed that women who had no history of neonatal death have an increased probability of knowing about preconception healthcare. The hypothesis to explain the possible reason for this may be due to parental stress and heightened anxiety because of the adverse outcome attributes [45]. In this case, the women may be at increased risk of an unplanned pregnancy because of an increased desire to have another baby. The current study finding is not consistent with a study performed in the Netherlands, Australia [46], and this might be due to study design differences, access to information and maternal health-seeking behaviours, and providers' counselling approach. Concerning family planning, women who used family planning methods were more knowledgeable. This finding is consistent with a study conducted in France [47], Gojjam, and Sudanese [25, 28]. Since planning a pregnancy is a vital component of PCH [48, 49], women who had access to family planning services might know about the risk of an unplanned pregnancy.

### 5.2. Limitations of This Study

The use of cross-sectional study design in this study may affect the causation for an alternative explanation of association. Self-reporting might have a social desirability bias. One of the limitations of this study was the lack of data on an obstetric parameter such as a delivery week, delivery mode, and birth weight. Given the limited factors addressed in this research, future research should consider healthcare providers and institutional-related factors [49].

## 6. Conclusion

The finding of this study shows a relatively satisfactory knowledge of preconception healthcare. In Jinka town, the probability of having good preconception healthcare increases when women become housewives, had formal education attendance, no history of neonatal death, and a history of family planning methods use. Therefore, the town health office needs to establish a preconception healthcare strategy in which it focuses on women's education and family planning use promotion. Moreover, women empowerment and knowledge improvement tailored to the community context involving essential stakeholders is mandatory.

## Figures and Tables

**Table 1:** Sociodemographic characteristics of the childbearing age group women in Jinka town, Southern Ethiopia, 2018 (*n* = 513).

Characteristics	Frequency	Percentage
Age		
15–24	259	50.5
25–34	176	34.3
35–49	78	15.2

Marital status		
Married	337	65.7
Single	135	26.3
Divorced	21	4.1
Widowed	20	3.9

Maternal occupation status		
Housewife	208	40.5
Civil servant	76	14.8
Merchant	53	10.3
Day labourer	28	5.5
Student	137	26.7
Others (private, jobless)	11	2.1

Maternal education status		
No formal education	68	13.3
Primary school	157	30.6
Secondary school (9–12)	155	30.2
College and above	133	25.9

Husband's education status		
No formal education	21	4.1
Primary school	85	16.6
Secondary school (9–12)	100	19.5
College and above	131	25.5%

Husband's occupation		
Government employee	135	26.3
Merchant	87	17.0
Day labourer	63	12.3
Farmer	19	3.7
Driver	18	3.5
Others	15	2.9

**Table 2:** Women's awareness of preconception healthcare in Jinka town, Southern Ethiopia, 2018 (*n* = 513).

Variables	Frequency (*N*)	Percentage (%)
Ever heard about preconception healthcare		
Yes	312	60.8
No	201	39.2

Source of information for preconception care		
Healthcare provider	254	49.5
Mass media	208	40.5
Friends	47	9.2
Others	4	0.8

Awareness on importance of preconception care		
Baby, only	38	7.4
Mother, only	43	8.4
Both baby and mother	380	74.1
Do not know	52	10.1

Convenient place for preconception care provision		
Home	56	10.9
Health institution	247	48.1
Home and health	148	28.8

Institution		
Do not know	62	12.1

**Table 3:** Women's knowledge of preconception health-related behavioural risk factors on the fetus in Jinka town, Southern Ethiopia, 2018 (*n* = 513).

Variables	Frequency (*N*)	Percentage (%)
Diabetes mellitus needs to be treated		
Yes	366	65.5
No	177	34.5

Epilepsy should be treated		
Yes	312	60.8
No	201	39.2

Uncontrolled obesity affects fetus health		
Yes	255	49.7
No	258	50.3

Screening for STI and HIV/AIDS improves fetal life		
Yes	412	80.3
No	101	19.7

Heart diseases should be treated		
Yes	319	62.2
No	194	37.8

Stress and depression affect fetal life		
Yes	263	51.3
No	250	48.7

Screening for genetic problem, lower adverse pregnancy outcome		
Yes	243	47.4
No	270	52.6

Cigarette smoking should be avoided before conception		
Yes	445	86.7
No	68	13.3

Alcohol consumption before conception result in poor pregnancy outcome		
Yes	426	83
No	87	17

Exposure to environmental hazard leads to adverse perianal outcome		
Yes	127	24.1
No	386	75.9

Illegal drug intake affects fetal wellbeing		
Yes	381	74.3
No	132	25.7

Female genital mutilation complicates childbirth		
Yes	382	74.5
No	131	25.5

**Table 4:** Factors associated with knowledge of preconception care among women in Jinka, Southern Ethiopia, 2018 (*n* = 513).

Variables		Knowledge of PCHC		Corollary (95% CI)	AOR (95% CI)
		Good	Poor		
Women's occupation	Housewife	121 (23.6%)	87 (17.0%)	1.26 (0.36–4.43)	2.93 (1.38–6.19)^*∗∗*^
	Civil servant	16 (3.1%)	60 (11.7%)	6.56 (1.71–25.2)	1.38 (0.72–2.63)
	Merchant	23 (4.5%)	30 (5.8%)	2.28 (0.60–8.75)	1.19 (0.56–2.74)
	Daily labourer	15 (2.9%)	13 (2.5%)	1.52 (0.36–6.37)	1.11 (0.60–2.1)
	Student	69 (13.5%)	68 (13.3%)	1.73 (0.48–6.16)	0.44 (0.11–1.67)
	Others	7 (1.4%)	4 (0.8%)	1 : 00	1 : 00

Women's education	No formal education	50 (9.7%)	18 (3.5%)	1 : 00	1 : 00
	Primary education	86 (16.8%)	71 (13.8%)	0.16 (0.08–0.	2.06 (1.06–3.97)^*∗*^
	Secondary education	75 (14.6%)	80 (15.6%)	0.36 (0.22–0.58)	2.77 (1.38–5.57)^*∗∗*^
	College and above	40 (7.8%)	93 (18.1%)	0.46 (0.28–0.75)	3.79 (1.75–8.23)^*∗∗*^

History of neonatal death	Yes	20 (3.9%)	5 (1%)	1 : 00	1 : 00
	No	139 (27.1%)	169 (32.9%)	4.86 (1.78–13.3)	4.13 (1.39–12.25)^*∗*^

History of family planning use	Yes	126 (24.6%)	178 (34.7%)	2.1 (1.47–3.01)	2.38 (1.49–3.79)^*∗∗∗*^
	No	125 (26.4%)	84 (16.4%)	1 : 00	1 : 00

^*∗*^, *p* value <0.05; ^*∗∗*^, *p* value <0.01; ^*∗∗∗*^, *p* value <0.001.

## Data Availability

The datasets generated during the current study are available from the corresponding author upon request.

## Abbreviations

CI: Confidence interval
LMIC: Low- and middle-income country
PCHC: Preconception healthcare
WHO: World Health Organization.
